# Progress in Medicine

**Published:** 1900-03-10

**Authors:** 


### Progress in Medicine.

### DISEASES OF THE KIDNEY.

**Renal Tuberculosis.**—The long duration of some of these cases is shown by the history of a patient who was seen by J. Frank[1]. She had suffered for 20 years, but the affection had been regarded as merely cystitis, and treated accordingly without success. Frank discovered bacilli in the urine, but in trying to find which kidney was at fault he failed entirely to pass a catheter into the ureter on the right side. However, by the use of Harris' separator it was made clear that the urine from the left was normal, while that from the right was deficient in urea, and showed pus and blood. He accordingly removed the right kidney, which proved to be the seat of long standing tuberculosis, and effected a cure. W. H. Battle[2] claims to have relieved a contracted bladder, though tubercular, by hydraulic distension. The patient suffered from tubercular lesions in various parts of the body, and complained grievously of the constant micturition. Boric acid solution was allowed to flow into the bladder from a height of 48 inches under an anæsthetic, which was omitted in the subsequent daily treatment. At first only four ounces could be introduced, but the amount rose to seven after a short time, and the power of holding the urine was some weeks later completely regained and other symptoms relieved. This case illustrates the dictum that the bladder is often without local lesions, though severely irritated by the passage of débris from the kidney. On the other hand, nearly all writers are agreed on the importance of avoiding all catheterisation or washing out the bladder in renal tuberculosis if it can possibly be avoided, since the mischief is most often aggravated by doing so. A critical review of the pathology and management of this disease is given by George Parker,[3] who argues that a great future is open to medical methods of treatment on the lines which have proved so successful with phthisis. Morris and Carmagny state that traces of the affection are found in one or two per cent. of all post-mortems. Recoveries are, therefore, not infrequent, and it is probable that a large percentage of cures could be obtained under rest, increased feeding, out-of-door treatment, together with the use of creasote, urotropin, sandal-wood oil, methylene blue, and other remedies. Bryson and Tilden Brown have shown instances of this. There are two forms of the disease—the descending, where the infection is carried from the blood stream to the cortex of the kidney, and only causes ulceration through the calices later on. Here, as Tilden Brown points out, there may be no symptoms for a long time. In the ascending form the infection passes upwards from the prostate epididymis and bladder to the kidney, and the patient's sufferings begin at an early stage. The symptoms include pain, continued or paroxysmal, in various parts of the urinary tract, bladder irritation, pus, and blood in the urine, which is at first acid, but later on alkaline and septic.

It contains from the beginning tubercle bacilli, while it is also increased in quantity. There may be a tumour or enlargement of the kidney, and usually, even at an early stage, a hard node is to be felt in the prostate. The hæmorrhage is not usually increased by exertion, and the bacillus can be distinguished from the smegma organism since the latter is decolorised by a bath in strong alcohol for about eight minutes. Bolton Bangs, in recommending nephrectomy, acknowledges a mortality of nearly 30 per cent., including those dying within nine months after the operation, or 20 per cent. of immediate deaths. The greatest importance must be attached to early diagnosis and vigorous anti-tubercular treatment.

David Newman[4] draws attention to the fact that renal tuberculosis sometimes follows a blow or fall, which has produced hæmaturia in a healthy person. He considers that the bacilli are normally excreted through a healthy kidney without affecting it, but if it suffers an injury tubercular lesions may be set up by the passing germs. He remarks, too, that hæmorrhage (from congestion) is at times the first sign of renal tuberculosis. In some cases years may elapse before other symptoms appear. In later stages hæmorrhage accompanies the breaking down of kidney tissue, and the blood is seen mixed with pus, débris, and albumen in turbid alkaline urine.

The prognosis of nephritis has been investigated by Cabot and White[5] from the history of 423 cases. They conclude that chronic nephritis, whether diffuse or interstitial, is not absolutely incurable, as tested by the absence of all subjective symptoms and the excretion of normal urine. The average duration of 332 cases was 19 months, but the disease may go on for many years without serious disturbance. Some of the cases due to lead, syphilis, or with a bad family history and no other observed causes, ran a very long course. Acute nephritis is a rarer disease than is generally thought. In fact, many supposed cases are merely exacerbations of chronic forms. Holst[6] thinks that a hæmorrhagic nephritis is often due to a general blood infection by strepto- or staphylococci, whether it has the appearance of an acute nephritis or an exacerbation. He gives cases showing sometimes an afebrile course and sometimes endocardial symptoms. Lindemann[7] recommends as a means of diagnosis the study of the freezing point of urine, which depends on the number of the molecules rather than on their weight. Thus in normal urine this point is nearly always between $-1{\cdot}2$ C. and $-2{\cdot}3$ C. If an inflammatory process exists in the kidneys the reduction of the freezing point is much less. Thus we can distinguish between interstitial and parenchymatous conditions. In the albuminuria of heart disease or cystitis there is no reduction, but if uræmia occurs in kidney disease the freezing point may be $-0{\cdot}7$ C. Early syphilitic nephritis is the subject of a study by Allaria,[8] who finds that when it occurs in the secondary stage it is marked by anasarca and severe albuminuria, and is cured under specific treatment. It is partly parenchymatous and partly interstitial. Tertiary forms are more chronic, and have less albumen.

Among symptoms of kidney disease H. W. Syers[9] notices dyspnœa pain and palpitation when accompanying hypertrophy of the left ventricle. For if the hypertrophy is due to valvular disease these sensations are absent, and the compensation lasts for a long while. He holds, too, that pericarditis is rare in renal disease, though apparently friction sounds can be heard. Other important diagnostic signs are the reduplication of the first sound at the apex and accentuation of the second at the base, especially when accompanied by dyspepsia. Tingling and numbness in the fingers, noises in the head, and peculiarities in the respiration may be the first symptoms to draw attention to the state of the kidneys. W. Carter,[10] in the Liverpool Journal, comments on the need of converting all the urinary poisons to the condition of urea. The sources of these poisons are the food (especially potash salts), retained secretions, substances absorbed from the intestines, and the products of tissue changes. Thus, besides diet and intestinal antisepsis, we may, by increasing oxidation, largely prevent kidney disorders. The cause of lardaceous degeneration has been investigated by Schepilewsky[11]. He confirms the view that it is produced very readily by the injection of large quantities of the staphylococcus aureus, yet he finds that substances which produce suppuration, but which are neither bacteria or their products, are also effective. The chemical cause of the change is something which is set free by the pus produced. Lardaceous degeneration is only a later stage of hyaline.

**Albuminuria.**—Eveno[12] discusses the formation and importance of nucleo-albumen, which was formerly mistaken in urine for mucin. It may depend on the action of toxins upon renal epithelium, or on some irritation affecting the genito-urinary tract. For its detection he dilutes the urine with a fourth of its volume of water, and adds acetic acid to precipitate it. He has observed it in ptomaine poisoning, in acromegaly, and after injections of creasote, as well as in bladder irritation. It is often mistaken for serum albumen, and may account for many cases of so-called functional albuminuria. The latter subject was discussed last year by West and Dreschfald. Among recent observations we may refer to those of Harold Williams,[13] who found albumen in the urine of every runner[13] after a 25 mile race, with casts in several cases, though the urines were quite normal beforehand, and became so again after two days. Teissier[14] holds orthostatic albuminuria, or that which only occurs when the patient is walking about depends on a local vascular congestion in certain neuroses, and is related to Raynaud's disease. The condition in a patient of his would appear in ten minutes after getting up. Coflin[15] has drawn attention to a form of albumen found especially in pregnant women, which is soluble in acetic and even in nitric acid, though precipitated by boiling without acidulation, or by trichloracetic acid or Tanret's reagent. H. P. Hawkins[16] divides albuminuria in the apparently healthy into that of exertion, neuroses, diet, and posture. The last is due to temporary injury of the renal epithelium from alterations in the blood pressure, or, in other words, to vaso-motor action. Such cases may remain for many years without any impairment of health, though it is doubtful whether the symptom ever disappears entirely. He would accept patients for insurance, but with a heavy addition to the premium.

Indican has been much referred to as an indication of excessive proteid decomposition in the intestines, or of suppurative peritonitis or pleuritis. Three methods of detecting it when present in abnormal quantities are recommended—the sulphuric acid and chloroform plan, the hydrochloric acid and hypochlorite of lime with chloroform plan, and Boardman Reed's.[17] The last is carried out by adding half a drachm of urine to a drachm of hydrochloric acid, and stirring them up. If there is an excess of indican a violet tint appears in a minute or two. One or two drops of strong nitric acid may be added if there is no reaction, and the tint of any indican present will be then seen.

1 Med. Rec., Sept. 9. 2 Lancet, Dec. 9. 3 Bristol Med. Chir. Jour., Sept. 4 Lancet, Aug. 26. 5 Med. Rec., Sept. 9. 6 Brit. Med. Jour., Oct. 14. 7 Med. Rec., Oct. 28. 8 Brit. Med. Jour., Dec. 30. 9 Treatment, Dec. 10 Med. Chronic., Oct. 11 Med. Chronic., Nov. 12 Treatment, Dec. 13 Practit., Sept. 14 La Semaine Méd., Dec. 20. 15 Philadel. Med. Jour., iii., 17, p. 957. 16 Brit. Med. Jour., Dec. 9. 17 Practitioner, Sept

### FEVERS.

(*Continued from page 309.*)

**Typhoid.**—Professor Wright's serum is stated[25] to consist of a pure culture of the bacillus which has been subjected to a temperature of 60 deg. Centig. Although it has only been in use a short time evidence is already at hand that the blood changes produced by it have lasted 18 months or two years; 2,855 troops were inoculated with it in India, and only 0·95 per cent. were afterwards attacked, with a mortality of 0·2 per cent., while of 8,000 uninoculated 2·5 were attacked and 0·34 per cent. died. Moreover, the inoculated were chiefly new arrivals in whom the ordinary rate is far higher, and it was not possible to institute two successive vaccinations for each man, so that the results really represent a high degree of protection, and a lessening of the severity of the disease in the few who do take it. The author calculates that even the present method would save 200 lives annually in the Indian army. A curious secondary result has also been noticed in the inoculated, for their liability to malarial fevers seemed to be greatly diminished.

Schäfer[26] finds that ulceration may occur in the fauces and on the palate during the early stages of typhoid. The favourite spot is the junction of the anterior pillars with the soft palate. The ulcers may be one or more in number, there is no false membrane, and rarely any enlargement of the glands or pain, and the ulcers heal up after some days without further complications. When present they may aid in the diagnosis of doubtful cases. Piorkowski[27] and others are able to obtain from the fæces cultures of the typhoid bacilli as early as the third day of the disease by using as a medium peptone gelatine mixed with normal urine. They think this method may even correct the Widal test. A very rare complication, hæmorrhagic purpura, is recorded by Musser and Sailer[28] in a young man who died after three weeks of typhoid. The petechiæ and hæmorrhages only occurred in the last five days. A few similar cases have been noticed by Osler, Nicholls, and others. A useful review of the question whether we are right in restricting typhoid patients to a strictly liquid diet is due to Mangés. He confesses that the evidence given by Barr, Bushuyev, Shattuck, and Fitz in favour of a fuller diet is after all too weighty to be neglected. One of them alone treated 318 patients in a year with success under this method, and the author shows that there is reason to doubt the validity of some of the usual arguments against it. He concludes that we are justified in cautiously trying the effect of more liberal diets modified to meet each individual case. R. W. Marsden,[29] of the Monsall Fever Hospital, has allowed some modification in the diet of 200 patients. All began, indeed, with milk, but mild cases went on to bread and milk, fish, mashed potato, chicken, bread, and minced meat, while a severe one might be kept to peptonised milk and milk juice. The patient's desire for food and the condition of his tongue were often taken as a guide, and practically half the number were on fish before defervescence was completed. There was no case of perforation, the percentage of hæmorrhages was small, and the diarrhœa and pyrexia did not seem to be influenced by the alteration of diet. On the other hand, he claims that recoveries were more rapid, and that asthenic complications were fewer. W. H. Thompson,[30] of the Roosevelt Hospital, analyses his results for 368 patients in 10 years, among whom he had a mortality of only 6 8 per cent., as against the 17 or 18 of English hospitals. He employs the cold bath with Nauheim salts, chiefly for its effect on elimination, feeds on milk and lime water with pepsin, and uses active purgation. F. E. Hare,[31] of Brisbane, who has reported such great results from cold baths, lays stress on the irregular effect of mere sponging from draughts, and consequent evaporation. Again, since obstinately high temperatures are not affected by sponging or tepid baths, he holds they should be treated with really *cold* baths, repeated and continued even for an hour at a time until they do succeed in bringing down the pyrexia. It is, fortunately, just these cases which support such vigorous treatment thoroughly well. The serum treatment has hitherto made little practical progress. From 1888 various antitoxins have been employed.[32] Cultures have been filtered, heated, or extracted with alcohol, and then injected into animals, and it has been shown that the serum of the latter has protective and curative powers. Klemperer and Chantemesse have gone on to treat typhoid patients with varieties of this serum raised to a very high degree of potency. The few cases about which reports have been received seem to have recovered from the disease in an incredibly short time, and to have escaped all the usual complications, so that it is hard to understand why no further trials of it have of late been made public.

[25] Brit. Med. Jour., Jan. 20. [26] Brit. Med. Jour., Jan. 6. [27] Lancet, Jan. 6. [28] Internat. Med. Jour., Nov. [29] Lancet, Jan. 13. [30] Med. Rec. [31] Australs. Med. Gaz. [32] Med. Rec., Dec. 9.

